# Basal shuttle of NF-κB/IκBα in resting T lymphocytes regulates HIV-1 LTR dependent expression

**DOI:** 10.1186/1742-4690-4-56

**Published:** 2007-08-08

**Authors:** Mayte Coiras, María Rosa López-Huertas, Joaquín Rullas, Maria Mittelbrunn, José Alcamí

**Affiliations:** 1 AIDS Immunopathology Unit, National Center of Microbiology, Instituto de Salud Carlos III, Majadahonda, Madrid, Spain; 2 Immunology Service, Hospital de La Princesa, Universidad Autonoma de Madrid, Madrid, Spain

## Abstract

**Background:**

In HIV-infected T lymphocytes, NF-κB/Rel transcription factors are major elements involved in the activation of LTR-dependent transcription from latency. Most NF-κB heterodimer p65/p50 is sequestered as an inactive form in the cytoplasm of resting T lymphocytes via its interaction with IκB inhibitors. In these cells, both absolute HIV latency and low level ongoing HIV replication have been described. These situations could be related to differences in the balance between NF-κB and IκBα ratio. Actually, control of IκBα by cellular factors such as Murr-1 plays a critical role in maintaining HIV latency in unstimulated T lymphocytes. Formerly, our group demonstrated the presence of nuclear IκBα in T cells after PMA activation. Now we attempt to determine the dynamics of NF-κB/IκBα nucleocytosolic transport in absence of activation as a mechanism to explain both the maintenance of latency and the existence of low level ongoing HIV replication in resting CD_4_^+ ^T lymphocytes.

**Results and conclusion:**

We show that the inhibition of the nuclear export by leptomycin B in resting CD_4_^+ ^T cells resulted in nuclear accumulation of both IκBα and p65/RelA, as well as formation of NF-κB/IκBα complexes. This proves the existence of a rapid shuttling of IκBα between nucleus and cytosol even in absence of cellular activation. The nuclear accumulation of IκBα in resting CD_4_^+ ^T lymphocytes results in inhibition of HIV-LTR dependent transcription as well as restrains HIV replication in CD_4_^+ ^T lymphocytes. On the other hand, basal NF-κB activity detected in resting CD_4_^+ ^T lymphocytes was related to low level HIV replication in these cells.

## Background

The nuclear factor κB (NF-κB) family of proteins are inducible transcription factors that play a central role in regulating the expression of a wide variety of genes associated with cell proliferation, immune response, inflammation, cell survival, and oncogenesis [1,2]. Functionally competent NF-κB is mainly composed by heterodimers of p65/RelA or c-Rel proteins complexed to p50/NF-κB1. NF-κB activity is regulated partially at subcellular level because active NF-κB heterodimers are normally sequestered in the cytoplasm via its non-covalent interaction with a family of inhibitory proteins termed IκBs, being IκBα the major NF-κB inhibitor protein. NF-κB activation is initiated by a variety of stimuli such as cytokines and growth factors, which lead to activation of IκB kinase complex (IKK). IKK in turn phosphorylates IκBα, resulting in its degradation via the ubiquitin-mediated proteolytic pathway. This permits NF-κB translocation into the nucleus, where engages cognate κB enhancer elements and modulates gene expression [1,2].

Control over NF-κB activity is not only accomplished through association with IκBα in the cytosol, but a role for nuclear IκBα in the control of NF-κB-driven transcription has been proposed [3,4]. In this model, newly synthesized IκBα would be able to shuttle actively between the cytoplasm and the nucleus, and then remove NF-κB from the -κB consensus sequences. Thus, nuclear IκBα would promote the return of NF-κB to the cytoplasm and the termination of its transcriptional response. The shuttle of NF-κB and IκBα between nucleus and cytosol in tumor cell lines has been described previously [3-5] as well as its influence on -κB dependent gene expression. However, in normal human CD_4_^+ ^T lymphocytes in a resting state, NF-κB binding activity is low and consists predominantly of inactive p50/p50 homodimers. In these cells, functional p50/p65 complexes are induced by cell activation [6]. Our group described previously that IκBα can translocate to the nucleus in T lymphocytes activated with phorbol-12-myristate-13-acetate (PMA) [7], but little is known about the existence of a NF-κB/IκBα shuttling in resting blood T cells.

The NF-κB pathway provides an attractive target to viral pathogens. Activation of NF-κB is a rapid, immediate early event that occurs within minutes after exposure to a stimulus, does not require *de novo *protein synthesis, and produces a strong transcriptional activation of several viral genes [6]. As a result, NF-κB is essential in the regulation of the HIV-1 long terminal repeat (LTR) promoter [8-10]. The promoter-proximal (enhancer) region of the HIV LTR contains two adjacent NF-κB binding sites (-109 to -79) that play a central role in mediating inducible HIV gene expression. These NF-κB responsive elements are major elements in triggering HIV LTR-transcription in blood CD_4_^+ ^T cells [6,9-11]. Accordingly, HIV production in T cells is mainly associated with the activation induced by different stimuli, whereas resting or unstimulated CD_4_^+ ^T lymphocytes offer a cellular environment for latency due to low permissiveness to HIV LTR activity [6]. However, the existence of a low-level ongoing replication in resting CD_4_^+ ^T lymphocytes has been described [12-14].

To reconcile these contradictory data, the hypothesis that the existence of a basal NF-κB activity could contribute to the low viral replication detected in HIV-infected CD_4_^+ ^T lymphocytes in a resting state is proposed. To this aim, the molecular mechanisms involved in the NF-κB/IκBα traffic between cytoplasm and nucleus of resting T lymphocytes from human blood have been analyzed. When resting CD_4_^+ ^T lymphocytes were cultured in presence of leptomycin B (LMB), a nuclear export inhibitor [15], both p65/RelA and IκBα were accumulated and associated in the nucleus, suggesting a rapid shuttling of both proteins in unstimulated T cells. In fact, HIV LTR-driven transactivation and HIV replication can be blocked in resting as well as activated T cells by IκBα over-expression. Our findings suggest that the balance between NF-κB and IκBα at nuclear level would be a key mechanism involved in both the maintenance of HIV latency and the induction of low-level HIV replication in resting CD_4_^+ ^T lymphocytes.

## Results

### Analysis of IκBα and p65/RelA subcellular distribution in resting CD_4_^+ ^T lymphocytes

Resting non-activated CD_4_^+ ^T lymphocytes were negatively isolated from human PBMCs by depletion of B cells, NK cells, monocytes, CD_8 _^+ ^T cells and activated lymphocytes. Analysis by flow cytometry revealed they were CD_4_^+ ^CD_25 _^- ^CD_69 _^- ^HLA-DR^- ^with a purity >95%.

**Figure 1:**
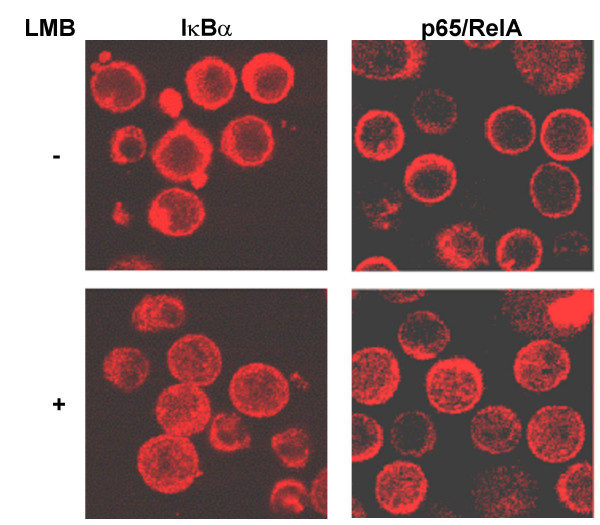
**Subcellular localization of IκBα and p65/RelA in CD_4_^+ ^T lymphocytes**. Cells were treated or not with 20 nM LMB and then fixed, permeabilized and stained with specific antibodies against IκBα and p65/RelA. A secondary antibody conjugated with Texas Red (Molecular Probes) was used. Images were taken by confocal microscopy.

IκBα and p65/RelA shuttling between nucleus and cytosol was analyzed in resting blood CD_4_^+ ^T cells by using LMB, a specific inhibitor of the nuclear protein export. The subcellular distribution of IκBα and p65/RelA was first analyzed by immunofluorescence assays. Both IκBα and p65/RelA were localized in the cytosol of unstimulated CD_4_^+ ^T cells (Fig. 1), but after treatment with LMB, both IκBα and p65/RelA were retained in the nucleus. This nuclear translocation was observed in the absence of any stimulus and was not due to serum activation since similar results were observed in serum deprivation conditions (data not shown).

**Figure 2:**
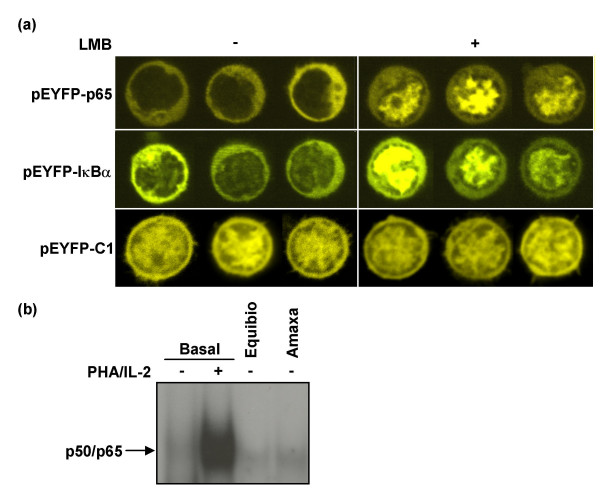
**Subcellular localization of EYFP-IκBα and EYFP-p65 fusion proteins in CD_4_^+ ^T lymphocytes**. (a) Cells were transiently transfected with 1 μg of either EYFP-IκBα or EYFP-p65 expression vectors per million of cells. LMB was added immediately after transfection. After 18–24 hours of incubation, cells were analyzed by confocal microscopy. pEYFP-C1 vector was used as control of unspecific distribution. (b) Resting purified CD_4_^+ ^T lymphocytes were transiently transfected with the control plasmid pcDNA3.1 by using an Amaxa nucleofector and a classical electroporator (Equibio). As occurs in untransfected resting T cells (lane 1), NF-κB was not induced in resting CD_4_^+ ^T lymphocytes after electroporation (lanes 3 and 4). As a positive control, NF-κB (p50/p65) binding was induced in these cells by PMA activation (lane 2).

These results were confirmed using chimeric proteins formed by the enhanced yellow fluorescent protein (EYFP) fused to IκBα or p65/RelA. Resting CD_4_^+ ^T cells were transiently transfected with plasmids pEYFP-p65 and pEYFP-IκBα separately. Analysis was performed 24 hours after transfection by confocal microscopy. There was low quantity of both IκBα and p65/RelA in the nucleus of the resting T cells before LMB treatment (Fig. 2a) but after exposure to LMB, both EYFP-IκBα and EYFP-p65 fusion proteins were retained in the nucleus. Plasmid pEYFP-C1 containing the EYFP under the control of CMV promoter was used as control of non-specific intracellular distribution.

To exclude that nucleoporation could induce NF-κB activity, electrophoretic mobility shift assays (EMSA) were performed in nuclear extracts from CD_4_^+ ^T lymphocytes transfected with a control plasmid (pcDNA3.1) by two different methods: the Amaxa Nucleofector system and classical electroporation using an Equibio electroporator (Figure 2b).

**Figure 3:**
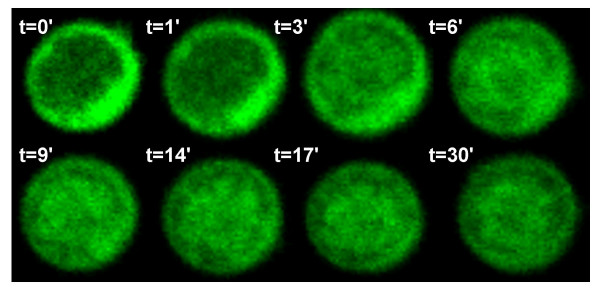
**Kinetic analysis of nuclear IκBα translocation**. One CD_4_^+ ^T lymphocyte transfected with EYFP-IκBα vector was photographed before and after treatment with LMB up to 30 minutes. Photographs were taken *in vivo *by confocal microscopy every minute after adding LMB.

In order to determine the dynamics of IκBα shuttling, resting CD_4_^+ ^T cells were transiently transfected with EYFP-IκBα vector, attached to fibronectine-coated slides and filmed *in vivo *by time-lapse confocal microscopy during treatment with LMB. Photographs were taken each minute after adding LMB and it was determined that less than 6 minutes were enough to saturate the nucleus with IκBα (Fig. 3 and additional file 1).

LMB toxicity was assessed by propidium iodide staining and flow cytometry in resting CD_4_^+ ^T cells treated up to 24 hours. Mortality due to LMB treatment (20 nM) was increased only 10% above controls after the longest incubation time (data not shown).

### Analysis of nuclear protein-protein interactions

Because more than 10^8 ^blood T lymphocytes for each experimental point were required to perform these experiments, T cells were expanded according to a protocol previously developed in our laboratory. PBMCs were cultured for 3 days with 5 μg/ml PHA and for the consecutive 9 days with 300 U/ml IL-2. These long-term cultures of PHA-treated T lymphocytes were maintained without supplemental IL-2 18 hours before the experiment to assure they were in a resting state concerning NF-κB activity. Following this protocol, it was proved that basal and induced NF-κB was similar as in resting T lymphocytes [7] (see Additional file 2).

**Figure 4:**
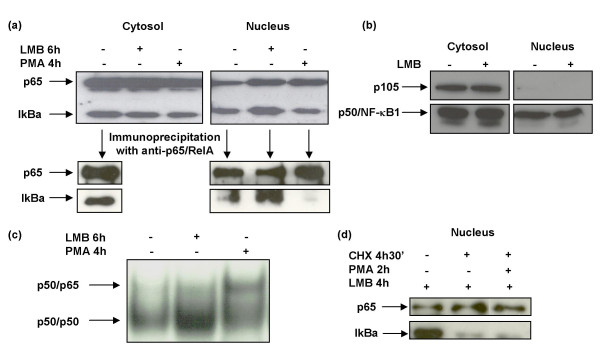
**Analysis of nuclear NF-κB/IκBα complexes in CD_4_^+ ^T cells and IκBα pool dependence on *de novo *protein synthesis**. (a) Analysis of subcellular distribution of p65/RelA and IκBα in CD_4_^+ ^T cells and presence of NF-κB/IκBα complexes in the nucleus after treatment with LMB or PMA. Ten micrograms of cytosolic and nuclear extracts from CD_4_^+ ^T cells treated with either PMA or LMB during 4 and 6 hours respectively were analyzed by Western Blot using antibodies against p65/RelA and IκBα. Immunoprecipitation assays were performed using 100 μg of these cytosolic and nuclear extracts, which were incubated with 5 μg of an antibody against p65/RelA conjugated with agarose. IκBα and p65/RelA complexes were characterized by immunoblotting. (b) Contamination with cytosolic proteins during nuclear protein extraction or accumulation of cytosolic proteins in the nucleus after treatment with LMB was assessed by Western Blot using an antibody against both p105 and p50/NF-κB1 proteins. (c) Analysis of NF-κB DNA-binding activity in CD_4 _^+^T cells treated with either PMA or LMB. Three micrograms of nuclear extract were incubated with an oligonucleotide containing the double consensus motif κB present in the HIV LTR labeled with [α-^32^P]-dCTP. Protein extracts were obtained from CD_4_^+ ^T cells after treatment with either LMB or PMA for 6 and 4 hours respectively. (d) Analysis of IκBα pool dependence on *de novo *protein synthesis. Ten micrograms of nuclear extracts from CD_4_^+ ^T cells incubated with 20 nM LMB for 4 hours and 10 μg/ml CHX and/or 25 ng/ml PMA for 4 hours,30 min and 2 hours, respectively, were analyzed by Western Blot.

Consequently, association between IκBα and p65/RelA was determined in the nucleus of long-term cultures of PHA-treated T lymphocytes. For this purpose, nuclear and cytosolic protein extracts were analyzed by immunoblotting assays. As previously shown for resting CD_4_^+ ^T lymphocytes (Fig. 1), both nuclear IκBα and p65/RelA levels increased in cells treated with LMB (Fig. 4a, Nucleus, lane 2). The accumulation of cytosolic proteins in the nucleus after LMB treatment has been ruled out by immunoblotting of cytosolic and nuclear extracts from PHA-treated T cells by using an antibody against both p105 and p50/NF-κB1 proteins (Fig. 4b). The p105 protein is the precursor of the p50 subunit and it presents exclusively a cytosolic location.

Immunoprecipitation assays with an antibody against p65/RelA showed the presence of NF-κB/IκBα complexes in the nucleus of T cells treated or not with LMB (Fig. 4a, Immunoprecipitation, Nucleus, lanes 1 and 2), whereas no association between p65/RelA and IκBα was observed in cells activated with PMA (Fig. 4a, Immunoprecipitation, Nucleus, lane 3).

### NF-κB DNA-binding activity in unstimulated T lymphocytes

Once it was confirmed that both p65/RelA and IκBα were able to shuttle between nucleus and cytosol in unstimulated T cells, NF-κB DNA binding activity was analyzed by EMSA. Despite the presence of p65/RelA in the nucleus, no binding was detected in unstimulated T cells treated or not with LMB (Fig. 4c, lanes 1 and 2). This correlated with the detection of NF-κB/IκBα complexes in the nucleus of these cells (Fig. 4a, Immunoprecipitation, Nucleus, lanes 1 and 2). As expected, NF-κB kept the binding activity to -κB motif in PMA-activated T cells (Fig. 4c, lane 3), due to the absence of NF-κB/IκBα complexes in the nucleus of these cells (Fig. 4a, Immunoprecipitation, Nucleus, lane 3).

### Analysis of IκBα resynthesis in resting T cells

Unstimulated T cells were incubated with CHX for 30 minutes before adding other stimulus in order to stop *de novo *protein synthesis. Then, LMB or PMA were added to the culture medium. Immunoblotting assays showed a decrease of IκBα levels in the nucleus of T cells incubated with both CHX and LMB (Fig. 4d, lane 2) or with CHX, LMB and PMA (Fig. 4d, lane 3), but not in those cells only incubated with LMB (Fig. 4d, lane 1). These data not only confirm previous results showing that nuclear translocation of IκBα is dependent on protein resynthesis [3] but also asserts that this *de novo *protein synthesis is carried out even in unstimulated T cells.

### Basal NF-κB activity can activate HIV-LTR promoter in CD_4_^+ ^T lymphocytes

**Figure 5:**
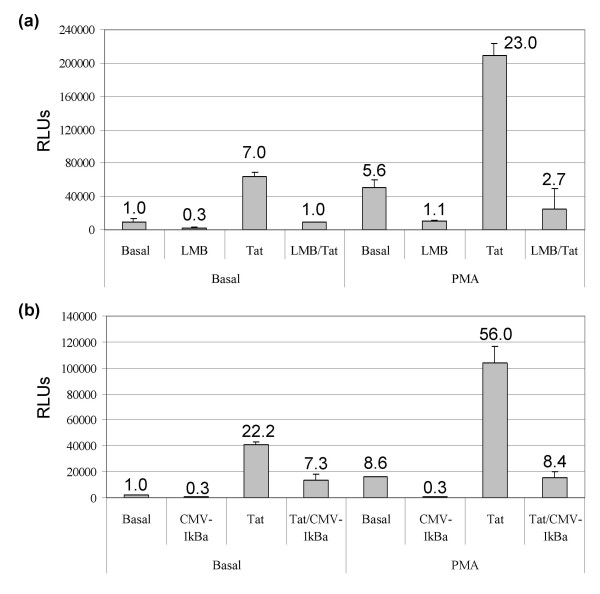
**Influence of IκBα over-expression on HIV-LTR transactivation**. Resting CD_4_^+ ^T cells were transfected with LTR-LUC vector together with (a) pcDNA3.1 and/or CMV-Tat expression vectors or (b) pcDNA3.1 and/or CMV-Tat and/or CMV-IκBα expression vectors, as indicated. Cells were treated with LMB immediately after transfection and/or with PMA two hours after transfection, as indicated. Luciferase activity was measured 18 hours after transfection. Numbers on the top of the bars represent fold transcriptional activity relative to unstimulated T cells transfected with pcDNA3.1.

Resting blood CD_4_^+ ^T cells were transfected with a LTR-LUC vector alone or together with a Tat expression vector under the control of the CMV promoter in order to assess NF-κB-dependent transcriptional activity in these cells by measurement of luciferase activity (Fig. 5a and 5b). As expected, both Tat over-expression and PMA activation enhanced LTR-dependent transcription, as previously described [11,16]. However, when nuclear levels of IκBα were increased by LMB (Fig. 5a) or transient transfection of CMV-IκBα vector (Fig. 5b), a dramatic decrease in luciferase activity was observed, both in PMA-activated T cells and cells in which Tat was over-expressed. Interestingly, a basal NF-κB activity able to induce a low LTR transactivation was detected in unstimulated CD_4_^+ ^T cells. This low LTR transactivation was annulled when IκBα was over-expressed by both LMB or CMV-IκBα transfection, thus proving this basal LTR transactivation was due to a residual NF-κB activity in resting CD_4_^+ ^T cells.

### Progression of HIV replication in resting CD_4_^+ ^T lymphocytes

**Figure 6:**
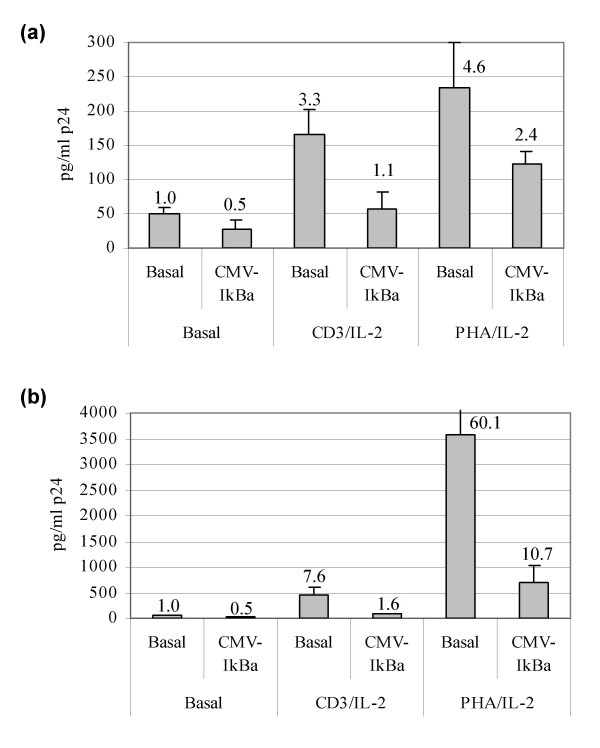
**HIV replication in resting or activated CD_4_^+ ^T cells transfected with an infectious molecular HIV-1 clone**. Highly purified CD_4_^+ ^CD_25 _^- ^CD_69 _^- ^DR^- ^T cells were transfected with the NL4.3 infectious molecular HIV-1 clone together with CMV-IκBα or pcDNA3.1 as negative control, and then activated with anti-CD_3 _and IL-2, PHA and IL-2, or maintained in the absence of activation. Viral replication was determined by quantification of HIV p24-gag antigen in culture supernatants (a) after 5 days of transfection or (b) after 7 days of transfection. Numbers on the top of the bars represent fold HIV-replication relative to unstimulated T cells transfected with pcDNA3.1. Differences in p24-gag production were significant for resting and anti-CD_3_-activated T cells (p < 0.05) and a trend towards statistical significance was found in PHA-activated T cells (p = 0.081).

To assess the role of basal NF-κB activity and IκBα over-expression on a model of HIV production in resting and activated CD_4_^+ ^T cells, highly purified CD_4_^+ ^CD_25 _^- ^CD_69 _^- ^DR^- ^T lymphocytes obtained from blood of different healthy donors were transfected with a full-length infectious HIV clone (NL4.3) together with a CMV-IκBα expression vector or pcDNA3 as negative control. Cells were maintained in culture up to 7 days either in the absence of activation or activated with two different stimuli, PHA and CD3 antibodies. HIV p24-gag was quantified 5 and 7 days after transfection. An intense HIV replication was detected in activated CD_4_^+ ^T cells after 7 days in culture (Fig. 6b). Besides, a discrete but significant HIV p24-gag production was assessed in resting CD_4_^+ ^T cells after 5 days of transfection (Fig. 6a). When IκBα was over-expressed in these cells, p24-gag production decreased as compared to cells transfected with a control plasmid and this difference was significant (p < 0.05) for resting and anti-CD_3 _activated T cells. Although more than five-fold decrease was observed at day 7 for PHA-activated lymphocytes when IκBα was over-expressed, this result did not reach statistical significance (p = 0.081).

## Discussion

Initiation of HIV transcription from a quiescent state is regulated through the concerted action of different cellular factors acting at LTR sequences [17,18]. Among them, NF-κB proteins are the most important inducible elements involved in initiation of HIV transcription in normal T cells [6,11,19-21]. As a result, a strong control of nuclear NF-κB translocation would be required to maintain HIV latency.

Nuclear translocation and activity of NF-κB is regulated through different mechanisms including association with its main inhibitor IκBα as a cytosolic inactive form. An additional mechanism of NF-κB control is the nuclear location of IκBα that act as a terminator of -κB dependent transactivation [4,5]. In fact, a dynamic shuttling of NF-κB has been described in established cell lines by balancing fluxes into and out of the nucleus [22-24] as well as the capacity of IκBα to enter the nucleus of T cells activated with PMA [7]. However, the nucleocytosolic shuttling of both NF-κB and IκBα in T cells in a resting state and its potential role in the maintenance of latency or the initiation of HIV transcription has not been determined so far. This is a very important issue, because resting CD_4_^+ ^T cells containing integrated HIV provirus constitute one of the long-lived cellular reservoirs of HIV *in vivo *[25,26] and represent a main obstacle to the eradication of the virus [27,28]. This HIV reservoir had been thought to be quiescent with regard to virus replication based on the principle that HIV production in T cells is linked to cellular activation. However, HIV production may occur in T cells that have not undergone classic T cell activation [29] and even in CD_4_^+ ^T lymphocytes lacking any activation markers [13].

These observations raise the question of whether NF-κB would be able to initiate the transcription of its target genes in resting T cells. In normal human CD_4_^+ ^T cells, NF-κB binding activity is low and consists predominantly of p50/p50 complexes, but not p50/p65. T-cell activation results in the formation of p50/p65 complexes and the induction of HIV-LTR transactivation. According to this hypothesis, both p65/RelA and IκBα showed a predominant cytosolic distribution in resting CD_4_^+ ^T cells (Fig. 1). However, a sharp increase in both nuclear IκBα and p65/RelA was found when nuclear export was inhibited by LMB, even in the absence of activation (Fig. 1 and 2). Moreover, *in vivo *kinetic studies determined that IκBα completely filled the nucleus of resting CD_4_^+ ^T cells in less than 6 minutes after adding LMB to the culture medium (Fig. 3 and additional file 1). NF-κB was associated to IκBα in the nucleus of resting T cells (Fig. 4a) and only p50/p50 heterodimers were able to bind DNA (Fig. 4c). In contrast, in PMA-activated T cells no association between IκBα and p65/RelA was found despite the presence of both proteins in the nuclear compartment (Fig. 4a), and consequently p50/p65 heterodimers could bind DNA (Fig. 4c). These results suggest the existence of post-translational modifications in p65/RelA and/or IκBα in PMA-activated T lymphocytes that would decrease the affinity between both proteins allowing DNA binding of active NF-κB. On the other hand, it has been described that only newly synthesized IκBα can enter the nucleus [4]. Accordingly, a sharp decrease in nuclear IκBα levels was observed in resting T cells when *de novo *protein synthesis was inhibited, whereas p65/RelA exhibited a longer half-life due to the existence of a pre-synthesized pool or a less active degradation (Fig. 4d). Therefore, a rapid degradation of IκBα occurs in T cells in the absence of activation and continuous synthesis is required to maintain a cytosolic pool of dissociated IκBα, able to translocate to the nucleus and capture NF-κB. These data proved not only the existence of a nucleocytosolic shuttling of IκBα and NF-κB in resting T lymphocytes but also that it is an extremely dynamic process detected exclusively when nuclear export is inhibited.

It has been described that HIV replication may occur within CD_4_^+ ^T cells activated below the threshold required for proliferation [12,13]. Indeed, it has been proposed that basal nuclear NF-κB translocation is required for the activation of genes involved in cell survival and these small discharges of nuclear NF-κB could be the cause of the low level replication observed in resting HIV-infected T cells. In support of this hypothesis, when CD_4_^+ ^T cells were transfected with luciferase expression vectors under the control of the HIV-LTR, low but consistent transcriptional activity, which was enhanced by Tat expression, was detected (Fig. 5a). In order to confirm that NF-κB was responsible for this low level LTR activity in resting T cells, nuclear levels of IκBα were increased by LMB (Fig. 5a) or transient transfection of CMV-IκBα vector (Fig. 5b). In both cases, LTR transcriptional activation decreased, even when Tat was also over-expressed. Moreover, despite the observation that in PMA-activated lymphocytes NF-κB was not bound to IκBα in the nucleus (Fig. 4a), IκBα over-expression resulted in strong decrease in HIV-LTR transactivation. It has been previously shown [3,4] that IκBα can bind p65/RelA and transport it back to the cytosol. When this pathway is blocked by LMB, IκBα cumulates in the nucleus at higher concentrations than during normal trafficking. We hypothesize that in these conditions NF-κB activity could be inhibited by high IκBα concentrations (Fig. 5). This observation supports that mechanisms involved in post-translational modifications of p65/RelA and/or IκBα induced by PMA, which block the formation of NF-κB/IκBα complexes, can be overcome by IκBα over-expression. Besides, low LTR transactivation detected in resting CD_4_^+ ^T cells was also annulled by IκBα over-expression, proving this basal LTR transactivation was due to a residual NF-κB activity in these cells.

To confirm the role of IκBα in an infectious model, a full-length proviral clone (NL4.3) was transfected in non-stimulated CD_4_^+ ^T cells together with a CMV-IκBα expression vector or pcDNA3.1 as negative control. This transfection method was used because the main goal was to analyze the role of IκBα over-expression on HIV replication in both resting and activated lymphocytes and classical infection models require previous T cell activation. In this system, low transfection rates of T lymphocytes are usually achieved but they were enough to induce full HIV replication after stimulation with PHA or anti-CD_3_. One open question in this model is whether p24-gag production derives from plasmid driven transient virus production and not yet full viral replication. Because T cell activation induces both HIV integration and further proviral transcription, full viral replication was achieved in PHA and anti-CD_3_-activated T lymphocytes. Moreover, increasing concentrations of p24-gag were detected throughout culture time, thereby suggesting several cycles of infection (Fig. 6). In this experimental system, inhibition of HIV replication by IκBα over-expression is probably produced during the first cycle of replication, because in subsequent replication cycles IκBα will not be over-expressed in non-transfected lymphocytes. Actually, a delay in HIV spread in culture due to partial inhibition of the first replication cycle in CMV-IκBα-transfected cells was observed (Fig. 6). Moreover, decrease in p24-gag production in CMV-IκBα-transfected cells was significant (p < 0.05) for resting and anti-CD_3 _activated T cells. Although for PHA-activated lymphocytes this difference was not significant, a five-fold decrease was observed at day 7 and a trend towards statistical significance was found (p = 0.081). On the other hand, it is difficult to precise if the mechanism involved in p24-gag production in non-activated T lymphocytes is due to plasmid-driven transient virus production and not yet to viral replication. However, our results showed a decrease in LTR transactivation (Fig. 5) and p24-gag production (Fig. 6) in resting CD_4_^+ ^T lymphocytes when IκBα is over-expressed. It suggests that increasing IκBα levels in naturally HIV-infected CD_4_^+ ^T lymphocytes carrying an integrated provirus could contribute to NF-κB inhibition and subsequent low-level viral production or absolute latency, as described in resting CD_4_^+ ^T lymphocytes *in vivo *[12-14,30,31].

On the other hand, it has been described that HIV can integrate into the genomes of in vitro-inoculated resting CD_4_^+ ^T cells that have not received activating stimuli [32]. Accordingly, HIV replication can also start in these cells although it cannot further progress unless these CD_4_^+ ^T cells were subsequently activated and NF-κB activity were maintained.

Overall, these data suggest that LTR transcriptional activation can be initiated by basal NF-κB activity in resting CD_4_^+ ^T cells in the absence of previous stimuli. Alternatively, the presence of high levels of nuclear IκBα would result in NF-κB control and viral latency. These data are supported by the existence of transdominant mutants of IκBα that block NF-κB induction and inhibit *de novo *HIV infection in T cells by interfering with viral transcription [20,33]. Besides, control of IκBα by other cellular factors such as Murr1, have been also involved in the maintenance of HIV latency in resting CD_4_^+ ^T lymphocytes [34].

## Conclusion

The maintenance of HIV latency should be considered an active cellular process. In resting CD_4_^+ ^T cells, both IκBα and NF-κB are continuously shuttling between cytosol and nucleus, as well as continuously associating and dissociating to permit a low transcriptional activity necessary for the activation of genes involved in cell survival. In resting HIV-infected T cells, the balance between free NF-κB and NF-κB/IκBα complexes in the nucleus could directly participate in the maintenance of HIV-latency when IκBα predominates as well as in the low ongoing HIV replication when NF-κB escapes IκBα control. Both phenomena have been characterized *in vivo *and constitute major pathogenic mechanisms in the persistence of long-lived cellular reservoirs of HIV [12-14,30,31]. Increased understanding of the control of NF-κB activation and repression would permit not only the development of new strategies to stop active HIV replication but also alternative treatments aimed at reactivation of latent HIV reservoirs in order to reduce them and contribute to viral eradication.

## Methods

### Cells

Peripheral blood mononuclear cells (PBMCs) were isolated from blood of healthy donors by centrifugation through a Ficoll-Hypaque gradient (Pharmacia Corporation, North Peapack, NJ). Cells were collected in supplemented RPMI and maintained at a concentration of 2 × 10^6 ^cells/ml. PHA-treated T lymphocytes were obtained from PBMCs incubated for 3 days with 5 μg/ml phytohemagglutinin (PHA) (Sigma-Aldrich, St. Louis, MO) and for the consecutive 9 days with 300 U/ml IL-2 (Chiron, Emeryville, CA). These long-term cultures of PHA-treated T lymphocytes were maintained without supplemental IL-2 18 hours before the experiment. These PHA-treated T lymphocytes remained at a pre-activated status and expressed activation markers [35] although NF-κB did not show DNA-binding activity (Additional file 2).

Resting CD_4_^+ ^T lymphocytes were isolated from PBMCs by negative selection with CD_4 _Negative Isolation Kit (T helper/inducer cells) (Dynal Biotech, Oslo, Norway), according to the manufacturer's instructions. Subsequently, isolated CD_4_^+ ^T cells were depleted of CD_25 _^+ ^by positive selection with Dynabeads CD_25 _(Dynal Biotech). Purity of isolated CD_4_^+ ^CD_25 _^- ^T cells was analyzed by flow cytometry with a FACScalibur flow cytometer (BD Biosciences, Erembodegem, Belgium). Cells were stained with monoclonal antibodies (mAb) against CD_4 _and HLA-DR conjugated with fluorescein isothiocyanate (FITC), and anti-CD_25_, -CD_69_, and -CD_3 _conjugated with phycoerythrin (PE), all provided by BD Biosciences. Analysis by flow cytometry revealed that the phenotype of isolated T lymphocytes was CD_4_^+ ^CD_25 _^- ^CD_69 _^- ^HLA-DR^- ^with a purity >95%.

### Reagents and antibodies

Cells were incubated with 25 ng/ml of 5-phorbol 12-myristate 13-acetate (PMA) (Sigma-Aldrich) for 30 min-18 hours. Leptomycin B (LMB) was used at 20 nM (Sigma-Aldrich). Cells treated with 10 μg/ml of cycloheximide (CHX) (Sigma-Aldrich) were incubated with this reagent 30 minutes before adding other stimuli. Primary antibodies against p65/RelA, p105/p50 and IκBα were obtained from Santa Cruz Biotechnology (Santa Cruz, CA). Secondary antibodies conjugated to horseradish peroxidase were purchased from GE Healthcare (Uppsala, Sweden). Secondary antibodies conjugated to Alexa 488 or Texas Red were purchased from Molecular Probes (Eugene, OR).

### Vectors

Luciferase reporter gene under the control of the U3+R regions of the HIV-long terminal repeat (LTR) (LAI strain) was previously reported [36]. pSV-β-Galactosidase vector (Promega, Madison, WI) was used to cotransfect the cells as an internal control reporter. IκBα gene cloned in pcDNA3.1(+) vector under the control of CMV promoter (CMV-IκBα) was described previously [37]. Viral Tat gene under the control of the CMV promoter (CMV-Tat) was also described previously [38]. pcDNA3.1(+) vector was used as negative control (Invitrogen, Carlsbad, CA). The vector pNL4.3 that contained the HIV complete genome and induced an infectious progeny after transfection in several cell lines was kindly provided by Dr M.A. Martin [39; National Institute of health AIDS Research and Reference Reagent Program #3418]. Dr. Johannes Schmid kindly provided the constructions of p65/RelA and IκBα genes in the enhanced yellow fluorescent protein vector (pEYFP-p65 and pEYFP-IκBα, respectively) [40,41]. Expression vector pEYFP-C1 (Clontech, BD Biosciences) that contains the yellow fluorescent protein gene under CMV promoter control was used as negative control. All plasmids were purified using Qiagen Plasmid Maxi Kit (Qiagen, CA), following the manufacturer's instructions.

### Transfection assays

CD_4_^+ ^T cells (5 × 10^6^) were transiently transfected with 2 μg of plasmid DNA under U-14 electroporation program conditions by nucleoporation with an Amaxa Nucleofector (Amaxa, Cologne, Germany) according to the manufacturer's instructions. Alternatively, CD_4_^+ ^T cells were also transfected by electroporation with an Easyjet Plus Electroporator (Equibio, Middlesex, UK). In brief, 10 × 10^6 ^cells were resuspended in 350 μl of RPMI without supplements and mixed with 1 μg of plasmid DNA per 10^6 ^cells in a 4 mm electroporation cuvette (Equibio). Cells were transfected at 320 V, 1500 μF and maximum resistance. After transfection, cells were incubated in supplemented RPMI at 37°C for 24 hours before analysis. Luciferase and β-Galactosidase activities were assayed using Luciferase Assay System and β-Galactosidase Enzyme Assay System, respectively, according to manufacture's instructions (Promega).

### Western blot assays

Cytosolic and nuclear protein extracts were obtained as described previously [7]. Ten micrograms of nuclear extracts were fractionated by sodium dodecyl sulfate-polyacrylamide gel electrophoresis (SDS-PAGE) and transferred onto Hybond-ECL nitrocellulose paper (GE Healthcare). After blocking and incubation with primary and secondary antibodies, proteins were detected with SuperSignal West Pico Chemiluminescent Substrate (Pierce, Rockford, IL).

### Immunoprecipitation assays

Cytosolic and nuclear protein extracts were subjected to immunoprecipitation with agarose-conjugated antibody against p65/RelA (Santa Cruz Biotechnology). In brief, nuclear or cytosolic proteins (100 μg) were incubated overnight at 4°C with 10 μg of specific agarose-conjugated antibody in RIPA buffer (PBS 1×, 0.1% SDS, 1% NP-40) and 0.5% sodium deoxycholate (DOC). Immunoprecipitate was collected by centrifugation at 4°C, 2.500 rpm for 5 minutes and washed four times with RIPA/DOC buffer. Finally, the agarose pellet was denatured at 95°C for 2 minutes and analyzed by SDS-PAGE, followed by immunoblotting with the specific antibodies.

### Electrophoretic mobility shift assays (EMSA)

Nuclear protein extracts (3 μg) were analyzed using the [α-^32^P]-dCTP-labeled double-stranded synthetic wild-type HIV enhancer oligonucleotide 5'-AGCTTACAAGGGACTTTCCGCTGGGGACTTTCCAGGGA-3' containing both κB consensus motifs. The nucleoprotein-oligonucleotide complexes were analyzed by electrophoresis on a non-denaturing 6% polyacrylamide gel.

### HIV replication assay

Resting CD_4_^+ ^T cells were transfected with pNL4.3 vector alone or with either CMV-IκBα or pcDNA3.1(+) vectors and further activated with 1 μg/ml anti-CD_3 _(BD Biosciences) plus 300 IU/ml IL-2. Viral replication was assessed by quantification of HIV p24 gag antigen in culture supernatants every 48 hours using an enzyme-like immunoassay (Innotest™ HIV Ag mAb, Innogenetics, Barcelona, Spain).

### Confocal microscopy

For immunofluorescence assays, cells were immobilized in PolyPrep slides (Sigma-Aldrich) for 15 minutes and then fixed with 2% paraformaldehyde-0.025% glutaraldehyde in PBS for 10 minutes at room temperature. After washing twice with 0.1% glycine/PBS, cells were permeabilized with 0.1% Triton ×-100/PBS for 10 minutes. After washing, cells were treated with 1 mg/ml NaBH_4 _for 10 minutes. Incubation for 1 hour at room temperature with each primary and secondary antibodies and subsequent washes were performed with PBS/2% bovine serum albumin (BSA)/0.05% saponine buffer. Coverslips were immobilized with 70% glycerol/PBS. Images were obtained with a Radiance 2100 confocal microscope (BioRad, Hercules, CA). For time-lapse fluorescence confocal microscopy, coverslips were coated with fibronectin (20 μg/ml) for 2 h at 37°C and blocked with PBS containing BSA 0.1%. Then, coverslips were washed with 1× Hanks Balanced Salt Solution (HBSS) and mounted in Attofluor open chambers (Molecular Probes). Cells were allowed to adhere on these chambers for 30 min. Confocal images were acquired using a Leica TCS-SP Confocal Laser Scanning Unit (Leica, Heidelberg, Germany) equipped with Ar and He-Ne laser beams and attached to a Leica DMIRBE Inverted Epi-Fluorescence Microscope. Images were processed and assembled into movies using Leica confocal software.

### Statistical analysis

Differences in HIV replication in the presence of IκBα over-expression were assessed by Mann-Whitney test using Statistical Product and Service Solutions (SPSS) software v14 (Addlink Software Científico, Madrid, Spain).

## Competing interests

The author(s) declare that they have no competing interests.

## Authors' contributions

MT carried out all the molecular biology studies and drafted the manuscript.

MRLH carried out the CD_4_^+ ^T cell isolation and performed the HIV replication assays.

JR and MM participated in the analyses by confocal microscopy.

JA conceived of the study, and participated in its design and coordination and helped to draft the manuscript.

All authors read and approved the final manuscript.

## Supplementary Material

Additional file 1 **Kinetic analysis of nuclear IκBα translocation**. Movie of one CD_4_^+ ^T lymphocyte transfected with EYFP-IκBα vector photographed before and after treatment with LMB up to 30 minutes. Photographs were taken *in vivo *by confocal microscopy every minute after adding LMB. This movie corresponds to Figure 3, which has been constructed with some pictures taken at different moments before and after adding LMB. It is an .avi file that can be viewed with Windows Media Player. Click here for file

Additional file 2 **Absence of NF-κB binding activity in nuclear protein extracts from unstimulated PHA-treated T cells**. (a) Binding of NF-κB in nuclear extracts from PHA-treated T cells to its cognate DNA sequence was analyzed. PBMCs were cultured for 3 days with 5 μg/ml PHA and for the consecutive 9 days with 300 U/ml IL-2. These long-term cultures of PHA-treated T lymphocytes were maintained without supplemental IL-2 18 hours. Three micrograms of nuclear extracts from IL-2 depleted T cells (lane 1) and activated with PMA for 30 min or 5 hours (lanes 2 and 3, respectively) were incubated with an oligonucleotide containing double -κB consensus motif from HIV LTR labeled with [α-^32^P]-dCTP. (b) Analysis of the NF-κB complexes composition by supershift assay. Three micrograms of nuclear extracts from PHA-treated T cells activated with PMA for 2 hours were incubated with antibodies against p50/NF-κB1 (lane 3), p65/RelA (lane 4) or c-Rel (lane 5) before the incubation with an oligonucleotide containing double -κB consensus motif from HIV LTR labeled with [α-^32^P]-dCTP. Lane 2 shows the specificity of binding of the NF-κB complexes using excess (100×) of unlabelled -κB-motif oligonucleotide as competitor. Click here for file
