# Supplementary figures and images for: Basal shuttle of NF-κB/IκBα in resting T lymphocytes regulates HIV-1 LTR dependent expression
Source: Retrovirology. 2007 Aug 8;4:56. doi: 10.1186/1742-4690-4-56 (PMC1988826; doi:10.1186/1742-4690-4-56)

## Slide 1
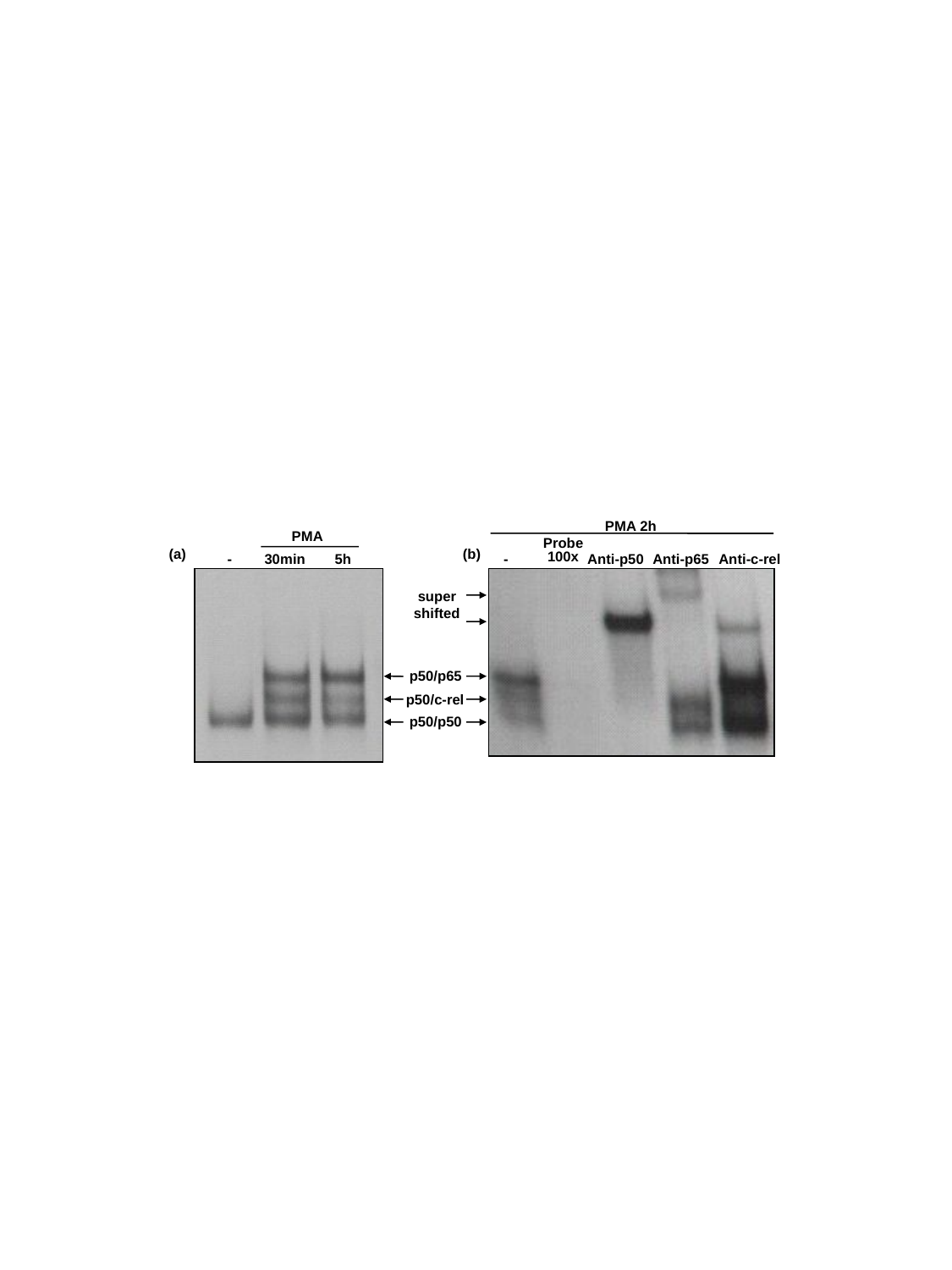

PMA 2h
PMA
Probe
100x
(a)
(b)
-
30min
5h
-
Anti-p50
Anti-p65
Anti-c-rel
super shifted
p50/p65
p50/c-rel
p50/p50

Supplement: Additional file 2 — Absence of NF-κB binding activity in nuclear protein extracts from unstimulated PHA-treated T cells. (a) Binding of NF-κB in nuclear extracts from PHA-treated T cells to its cognate DNA sequence was analyzed. PBMCs were cultured for 3 days with 5 μg/ml PHA and for the consecutive 9 days with 300 U/ml IL-2. These long-term cultures of PHA-treated T lymphocytes were maintained without supplemental IL-2 18 hours. Three micrograms of nuclear extracts from IL-2 depleted T cells (lane 1) and activated with PMA for 30 min or 5 hours (lanes 2 and 3, respectively) were incubated with an oligonucleotide containing double -κB consensus motif from HIV LTR labeled with [α-32P]-dCTP. (b) Analysis of the NF-κB complexes composition by supershift assay. Three micrograms of nuclear extracts from PHA-treated T cells activated with PMA for 2 hours were incubated with antibodies against p50/NF-κB1 (lane 3), p65/RelA (lane 4) or c-Rel (lane 5) before the incubation with an oligonucleotide containing double -κB consensus motif from HIV LTR labeled with [α-32P]-dCTP. Lane 2 shows the specificity of binding of the NF-κB complexes using excess (100×) of unlabelled -κB-motif oligonucleotide as competitor. [file 1742-4690-4-56-S2.ppt]
